# Characteristics of private health insurance enrollees and the association between health coverage type and outpatient service utilization in Saudi Arabia: Insights from the National Saudi Family Health Survey

**DOI:** 10.1371/journal.pone.0312887

**Published:** 2026-04-30

**Authors:** Khaled Shaeel Althabaiti, Monica Hunsberger, Jahangir Khan, Sayem Ahmed

**Affiliations:** 1 School of Public Health and Community Medicine, Institute of Medicine, Sahlgrenska Academy, University of Gothenburg, Gothenburg, Sweden; 2 Basic Nursing Sciences Department, College of Nursing, Taif University, Taif, Saudi Arabia; 3 Department of International Public Health, Liverpool School of Tropical Medicine, Liverpool, United Kingdom; 4 Department of Learning, Informatics, Management and Ethics, Karolinska Institutet, Stockholm, Sweden; 5 Health Economics Research Group (HERG), Department of Health Sciences, Brunel University London, Uxbridge, London, United Kingdom; University of Bisha, SAUDI ARABIA

## Abstract

The Kingdom of Saudi Arabia (KSA) has a mixed health financing system consisting of governmental health coverage (GHC) and private health insurance (PHI). In 2016, KSA launched Vision 2030, which aims to reduce government spending and increase reliance on employer-sponsored PHI. This reform may affect the utilization of health services based on citizenship and the type of health coverage (PHI compared with GHC). The current study aimed to identify the characteristics of private health insurance enrollees and the association between different types of health coverage (GHC and PHI) and outpatient service utilization in the KSA. This study used data from the 2018 Saudi Family Health Survey (SFHS), which included 8,276 respondents aged 18 years and above and collected information on outpatient utilization during the previous 12 months. Statistical analyses were conducted using SPSS version 26. Bivariate analyses (chi-square and t-tests) were used to assess differences by nationality and coverage type. Binary logistic regression was used to examine the characteristics of PHI enrollment, and Poisson regression was used to evaluate the association between coverage type and outpatient utilization. This dataset provides a pre-reform baseline for assessing PHI enrollment and outpatient utilization prior to the implementation of the first phase of the reform in 2019. Most respondents were Saudi nationals (76.8%), and 54.9% were male. About 26.2% of respondents had PHI. Among non-Saudis, 72.8% were enrolled in PHI, compared with only 12.1% of Saudis. The logistic regression analysis revealed that having PHI was associated with factors such as a high monthly income, non-Saudi, male, being married, a high level of education, and a perception of good health. We found that having PHI was negatively associated with the utilization of outpatient services (coefficient −0.107; P < 0.001) compared with GHC. This finding may reflect barriers to access, a lower perceived need, or limitations in awareness of PHI benefits. In addition, this finding suggests that PHI may be associated with disparities in healthcare access, based on the observed lower utilization of outpatient services among its enrollees compared with those covered by GHC. Other factors associated with the utilization of outpatient services were being female, having a high monthly income, being never married, having chronic diseases, and the perception of poor health. The results of this study may inform future health reform efforts to enhance access to healthcare. These findings should be considered when planning the implementation and evaluation of the health system in KSA.

## Introduction

The Kingdom of Saudi Arabia (KSA) currently provides healthcare services free of charge to its citizens under the governmental health coverage (GHC) [1]. Non-Saudis working in the governmental sector have similar access to healthcare under GHC. Before 1999, all residents received free services in public healthcare facilities regardless of their employment status or nationality. However, the government recognized that the current healthcare model faces long-term financial challenges due to several factors, including population growth and increasing demand for healthcare services. This demand increases government healthcare spending and contributes to overcrowding in public facilities, and both financial and operational pressure on the healthcare system, necessitating reforms to ensure sustainability and equity [2,3]. This recognition led to a shift in policy direction, as Saudi government policymakers began the transition to an employer-mandated insurance scheme under private health insurance (PHI) through the Cooperative Health Insurance Law, enacted in 1999. From 2006, this scheme was fully implemented for expatriates only in the private sector [4]. The scheme aimed to relieve the financial burden on the public sector by requiring private sector employers, which form 67.9% of the workforce in Saudi Arabia, to provide PHI for their Saudi (22.3%) and non-Saudi (77.7%) employees. This scheme will encourage private sector employees (Saudis and expatriates) to utilize private healthcare providers for their health services [5,6].

In KSA, the Ministry of Health (MOH) is the main public authority responsible for the health system, while healthcare services are delivered through MOH facilities, other governmental sectors such as the National Guard and educational health facilities, and private healthcare providers [7]. According to reports published by the MOH in 2022, the number of visits to the healthcare facilities in the public sector (hospitals and primary healthcare centers) accounted for 78.6 million (61%), while it reached 20.4 million (16%) in other governmental sectors and 29.5 million (23%) in the private sector [8,9]. Over the past few decades, KSA has witnessed rapid growth in healthcare investments, including hospitals that provide secondary and tertiary care, as well as large hospitals offering comprehensive services [10].

In recent years, KSA has expanded its insurance-based healthcare financing by mandating PHI not only for private-sector employees but also for tourists, pilgrims, and domestic workers. This policy shift aimed to reduce the strain on publicly funded healthcare services and promote greater private sector engagement. This strategy offers medical coverage to more than 11 million insured people in KSA through 27 private insurance companies [11]. In 2016, KSA launched Vision 2030, a strategic plan aimed at reforming the economy and key sectors, including health, education, tourism, and infrastructure. In healthcare, the focus is on improving quality, accessibility, and equity by increasing private sector involvement and ensuring universal health coverage (UHC) for all residents; these reforms are implemented in three phases. The first phase included coverage for employees of large companies employing 500 or more individuals, gradually extending to smaller businesses, and eventually covering all employees, including Saudis and non-Saudis in the private sector, under an employer-mandated insurance scheme. This first phase of the reform was fully implemented in 2019 [12–14]. The second phase covers those working in the government sector under the National Health Insurance program. The third phase will cover all citizens regardless of their work or status [15]. Currently, the reform is in its second phase, while the remaining stages are still pending.

There are two types of health coverage in KSA: firstly, the GHC, which provides health services in public facilities to the Saudi population and non-Saudis working in the government sector [16]. Secondly, PHI, which includes employer-sponsored insurance as one of its main forms, where the employer in the private sector takes responsibility for the payment of enrollment and renewal of PHI for employees, including both citizens and expatriates [17]. According to the General Authority for Statistics in 2023, this PHI covered 37.5% of the total adult population aged 15 and above (20.5% for citizens and 53.6% for expatriates) [18]. In addition, PHI is available to citizens and expatriates through individual purchase to extend their health services benefits, as well as international students, tourists, and pilgrims who must be covered by the PHI before their entrance to the country [19].

Previous research in KSA showed that individuals with PHI had higher utilization of health services than those without PHI, especially for routine check-up services [20], periodic preventive services [11], inpatient and outpatient services, visits to the emergency room (ER) [21], and dispensing medicines [22]. Alrabiah and colleagues found that PHI increased adherence to treatment and a positive perception of the quality of received services [23]. Moreover, research indicated that individuals with PHI utilized outpatient services more frequently due to easier accessibility and the availability of a wide range of medical services [24]. However, Saudi Arabia has a large expatriate population including both skilled and unskilled workers and their dependents [2] yet existing studies lack sufficient exploration of the discrepancies between Saudis and non-Saudis regarding enrollment in PHI and utilization of healthcare services. A study that includes more unexplored factors can provide a more comprehensive understanding of the effect of PHI on the utilization of health services by various demographic groups [20]. By informing policy development, such insights can lead to targeted interventions aimed at improving health or health insurance literacy and increasing insurance coverage [25], and in the long term, this may enhance health outcomes. In light of the ongoing healthcare system reforms in KSA, research is needed to pinpoint areas that require improvement and guide the development of more effective policies to enhance healthcare utilization and overall health outcomes in the country [26].

The present study primarily focuses on PHI, which includes both employer-provided insurance and insurance purchased voluntarily by employees or non-employees of Saudi citizens. In 2018, this PHI was mandatory for expatriates working in the private sector and optional for Saudi citizens, thereby providing a pre-reform baseline. In addition, the study considers GHC, which covers Saudi citizens and non-Saudis working in the government sector. This research is driven by the need to understand the relationship between PHI enrollment and outpatient service utilization in KSA, particularly within the recent healthcare reforms, which focus on shifting from government-funded healthcare to PHI, primarily targeting expatriates. However, our data indicate that this shift had not yet been fully implemented in 2018. This transition addresses the rising demand for medical services due to population growth and a significant expatriate community, and should also focus on health insurance literacy among workers and individuals [6,27]. Understanding the factors influencing PHI enrollment is crucial for policymakers to design effective interventions that promote increased insurance coverage and utilization of outpatient services. However, previous studies mainly compared individuals with PHI to those without PHI and did not explicitly examine differences in healthcare utilization between individuals with PHI and those assumed to rely on government-provided healthcare. This study addresses this gap using data from the 2018 Saudi Family Health Survey.

This study aims to identify the characteristics of PHI enrollees and the association between different types of health coverage and outpatient service utilization in KSA.

## Materials and methods

This research employed a cross-sectional study design using data from the 2018 Saudi Family Health Survey (SFHS), which provides a pre-reform snapshot of PHI enrollment and outpatient service utilization in KSA. This dataset provided a valuable reference point for analyzing PHI enrollment and outpatient service utilization prior to the 2019 reforms. The SFHS was a household survey classified under education and health statistics conducted by the General Authority for Statistics (GASTAT). This survey comprises a total of 12,827 respondents, collected from all 13 administrative areas in KSA. The final analytical sample for this study consisted of 8,276 individuals aged 18 years and above. The survey used a pre-designed, validated questionnaire that had been adopted from the World Health Organization (WHO)’ World Health Survey (WHS).

The survey contained questions about the household’s demographic characteristics of the participants (age, sex, nationality, education level, marital status, number of family members, monthly income, employment status, and residence), health status (self-rated health condition and any chronic diseases), and the type of health coverage (GHC or PHI). The PHI was defined broadly to include both employer-sponsored PHI and individually purchased PHI. Employer-sponsored PHI is typically linked to employment in the private sector and financed through employer-paid premiums, whereas individually purchased PHI is directly funded by the individual. GHC is government-financed and provides access to health services delivered through public-sector health facilities for citizens and expatriates working in the public sector.

Trained data collectors conducted the interviews using the pre-designed questionnaire [28]. The data from the survey were officially requested from the sponsor of the data in the government of Saudi Arabia, represented by GASTAT. The authority provided the data in Excel format that included the relevant variables for the current study. The data were translated into English and coded to facilitate statistical analysis using the SPSS ver. 26 statistical package.

### Data analysis

The descriptive statistics presented frequency, mean, and standard deviation. The chi-squared test was used to evaluate the association between participants’ characteristics and type of health coverage. An independent sample t-test was conducted to investigate the significance of the difference in the number of medical facility visits between individuals with GHC and PHI. We employed multiple regression analysis in two models (separately): one predicting “private health insurance enrollment” and the other predicting “Healthcare Utilization”. In the first model, the independent variables included chronic disease, perceived health status, nationality, sex, monthly income, education level, age, and marital status.

In the first model, we used binary logistic regression to identify which factors (characteristics) significantly predicted private health insurance enrollment. In the second model, Poisson regression was employed to determine if PHI enrollment is associated with healthcare service utilization compared with the GHC. Overdispersion was assessed before model selection and was not substantial; therefore, Poisson regression was retained. In this model, we controlled for several variables, like chronic disease, perceived health status, nationality, sex, monthly income, education level, age, and marital status.

### Variables selection

We used the Andersen Behavioral model as the guiding theoretical framework to identify factors influencing healthcare utilization under three categories: predisposing factors (age, sex, education level, marital status, and nationality), enabling factors (monthly income and type of health coverage), and need factors (health condition and chronic disease status). This model assessed how sociodemographic characteristics and types of health coverage are associated with healthcare utilization [29].

We conducted multicollinearity diagnostics by calculating the Variance Inflation Factor (VIF) for each independent variable using a linear regression model in the SPSS program. The VIF values were transformed into Generalized Variance Inflation Factor (GVIF) due the independent variables were categorical. The following formula was applied:


$$\text{GVIF}={\text{VIF}}^{\wedge }\left(\text{1}/(\text{2}*\text{df})\right)$$

where df represents the degrees of freedom of the categorical variable. The (GVIF < 5) was calculated to ensure no multicollinearity among the categorical independent variables [30].

Since SPSS does not provide built-in diagnostics for multicollinearity in Poisson or logistic regression models, a linear regression framework was used for this assessment [31].

### Ethical clearance

This study utilized secondary data from the SFHS, which was conducted, commissioned, funded, and managed in 2018 by GASTAT, which was responsible for all ethical procedures. We obtained official permission from GASTAT to use the dataset. Additionally, ethical clearance was obtained from the Swedish Ethical Review Authority (Registration number: 2025-03265-01).

## Results

Table 1 presents the demographic and socioeconomic characteristics of the respondents, categorized according to nationality. Out of all respondents (n = 8,276), 76.8% were Saudi nationals. The proportion of the male population was higher among non-Saudis (69.1%) than the Saudis (50.6%). The majority of respondents (55.8%) were aged between 26 and 59 years. The respondents from this age group comprised 78.8% of non-Saudis and 58.7% of Saudis. Only 22.3% of total respondents had higher education, either a university qualification or a postgraduate degree. Approximately 23.3% of Saudis and 19.1% of non-Saudis had such educational qualifications.

**Table 1:** Demographic characteristics of the study participants (n = 8,276).

Characteristics		Total		Saudi		Non-Saudi		P^a^
		N (8,276)	%	N (6,351)	%	N (1,925)	%	
**Sex**	Male	4,545	54.9%	3,215	50.6%	1,330	69.1%	<0.001**
	Female	3,731	45.1%	3,136	49.4%	595	30.9%	
**Age**	18-25 years	1,912	23.1%	1593	25.1%	319	16.5%	<0.001**
	26-59 years	5,248	63.4%	3732	58.7%	1,516	78.8%	
	>60 years	1,116	13.5%	1,026	16.2%	90	4.7%	
**Family monthly income (Saudi Riyal)**	<5,000	2,580	31.2%	1,433	22.6%	1,147	59.6%	<0.001**
	5000- < 10000	2,901	35.1%	2,415	38.0%	486	25.2%	
	10000- < 15000	1,416	17.1%	1,280	20.2%	136	7.1%	
	15000- < 20000	681	8.2%	612	9.6%	69	3.6%	
	20000+	698	8.4%	611	9.6%	87	4.5%	
**Education level**	Below primary school	1,401	16.9%	1,052	16.6%	349	18.1%	<0.001**
	Primary school	753	9.1%	495	7.8%	258	13.4%	
	Intermediate school	1,008	12.2%	651	10.3%	407	18.5%	
	Secondary school	3,270	39.5%	2,675	42.1%	595	30.9%	
	Higher education	1,844	22.3%	1,478	23.3%	366	19.1%	
**Marital status**	Married	5,361	64.8%	3,948	62.2%	1,413	73.4%	<0.001**
	Never married	2,463	29.7%	1,986	31.3%	470	24.4%	
	Divorced & Widowed	459	5.5%	417	6.6%	42	2.2%	
**Working Status**	Employed	3,969	48.0%	2,507	39.5%	1,462	75.9%	<0.001**
	Unemployed	1,067	12.9%	999	15.7%	68	3.6%	
	Housewife	2,493	30.1%	2,104	33.1%	389	20.2%	
	Retired	747	9.1%	741	11.7%	6	0.3%	
**Health coverage types**	Private health insurance	2,170	26.2%	768	12.1%	1402	72.8%	<0.001**
	Governmental health coverage	6,106	73.8%	5,583	87.9%	523	27.2%	
**Types of private health insurance (N = 2,170)**	Employer-Sponsored Private Health Insurance	2044	94.2%	743	96.7%	1301	92.8%	<0.001**
	Individually Purchased Private Insurance	126	5.8%	27	3.3%	99	7.2%	
**Self-rating of health status**	Very good	5,933	71.7%	4,354	68.5%	1,579	82.0%	<0.001**
	Good	1,504	18.2%	1,219	19.2%	285	14.8%	
	Fair	642	7.8%	596	9.4%	46	2.4%	
	Poor	197	2.4%	182	2.9%	15	0.8%	
**Have chronic disease**	Yes	2,108	25.5%	1,830	28.8%	274	14.4%	<0.001**
	No	6,168	74.5%	4,521	71.2%	1,647	85.6%	
**Type of chronic disease (n = 2,108):**	Diabetes	702	33.3%	600	32.8%	102	36.7%	0.046**
	Hypertension	630	29.9%	557	30.4%	73	26.2%	
	Musculoskeletal	124	5.8%	113	6.2%	11	4.0%	
	Hypercholesterolemia	100	4.7%	81	4.4%	19	6.8%	
	Cardiovascular	97	4.6%	88	4.8%	9	3.2%	
	GIT	67	3.1%	64	3.5%	3	1.1%	
	Bronchial asthma	60	2.8%	53	2.8%	7	2.5%	
	Others	328	15.8%	274	15.1%	54	19.5%	
**Number of healthcare outpatient visits in past 12 month:**	0	2,122	25.6%	1,214	19.2%	908	47.3%	<0.001**
	1	2,446	29.6%	1,883	29.6%	563	29.2%	
	2	1,959	23.7%	1,709	26.9%	250	13.0%	
	3	993	12.0%	847	13.3%	146	7.5%	
	4	383	4.6%	360	5.7%	23	1.2%	
	5+	373	4.5%	338	5.3%	35	1.8%	

Almost half of the respondents (48.0%) were employed, with a higher percentage among non-Saudis (75.9%) than Saudis (39.5%). Regarding the monthly income, a higher proportion of non-Saudis (59.6%) than Saudis (22.6%) had the lowest monthly income (<5,000 SR). Overall, (64.8%) of the respondents were married, with a higher percentage of non-Saudis (73.4%) than Saudis (62.2%). Regarding types of health coverage, respondents were classified into two groups: PHI (26.2%), and GHC (73.8%). Further, the types of PHI included: employer-sponsored PHI (94.2%) and individually purchased PHI (5.8%). PHI enrollment was higher among non-Saudis (72.8%) compared with Saudis (12.1%). The majority of the Saudis (87.7%) and non-Saudis (96.8%) perceived their health status as good or very good. Chronic diseases (mostly diabetes and hypertension) were reported by 28.8% of the Saudis and 14.4% among non-Saudis. At least one healthcare visit was reported in the past 12 months by 80.9% of Saudi respondents and 52.8% of non-Saudis.

Table 2 presents the proportion of respondents enrolled in PHI according to their characteristics. The PHI enrollment was significantly associated with the sex of the respondents, with a higher rate of enrollment among male (32.5%), compared with females (18.5%). A similar observed difference in PHI enrollment between male and female from the non-Saudi and Saudi groups. The PHI enrollment was highest in those who were aged 26–59 years (31.4%). The employed had a higher PHI enrollment rate than the unemployed (36.4% vs 12.4%). It was observed that the percentage of the PHI was significantly higher among those with lower monthly income <5,000 (36.5%), which may reflect mandatory coverage for low-income expatriate workers. Among Saudis and non-Saudis, the highest percentage of PHI enrollment was for the income group 5000- < 10000 and 15000- < 20000, respectively. Marital status, education level, and chronic disease were significantly associated with the PHI enrollment.

**Table 2:** Comparing the private health insurance enrollment rate of the Saudi and non-Saudi populations according to their characteristics.

Characteristics		Private health insurance-Saudi			Private health insurance-non-Saudi			Private health insurance-all		
		n (6,351)	%	P-value*	n (1,925)	%	P-value*	N (8,276)	%	P-value*
Sex	Male	3,215	13.3	0.004	1,330	79.2	<0.001	4,545	32.5	<0.001
	Female	3,136	10.9		595	58.7		3,731	18.5	
Age	18-25 years	1,593	9.5	<0.001	319	65.5	0.002	1,912	18.8	<0.001
	26-59 years	3,732	13.6		1,516	75.2		5,248	31.4	
	>60 years	1,026	10.6		90	58.9		1,116	14.5	
Education	Below primary school	1,052	5.8	<0.001	349	58.5	<0.001	1,401	18.9	<0.001
	Primary school	495	7.1		258	61.6		753	25.8	
	Intermediate school	651	11.7		357	67.5		1,008	31.4	
	Secondary school	2,675	13.9		595	78.8		3,270	25.7	
	Higher education	1,478	15.2		366	89.9		1,844	30	
Monthly income (Saudi Riyal)	<5000	1,433	7	<0.001	1,147	73.4	<0.001	2,580	36.5	<0.001
	5000- < 10000	2,415	10.3		486	79.2		2,901	21.9	
	10000- < 15000	1,280	14.1		136	68.4		1,416	19.3	
	15000- < 20000	612	21.6		69	50.7		681	24.5	
	20000+	611	17.5		87	54		698	22.1	
Chronic disease	Yes	1,830	12.7	0.32	278	68	0.05	2,108	20	<0.001
	No	4,521	11.8		1,647	73.6		6,168	24.2	
Working Status	Employed	2,507	14.5	<0.001	1,462	73.9	0.136	3,969	36.4	<0.001
	Unemployed	999	8.2		68	73.5		1,067	12.4	
	Housewife	2,104	10.4		389	68.9		2,493	19.6	
	Retired	741	13.9		6	50		747	14.2	
Marital status	Married	3948	13.9	<0.001	1413	76.6	<0.001	5361	30.4	<0.001
	Never married	1986	9.8		470	64.5		2456	18.5	
	Divorced & Widowed	417	6		42	40.5		459	9.2	
Self-rating of health status:	Very Good	4,352	12.2	<0.001	1,579	72.9	0.002	5,931	28.4	<0.001
	Good	1,219	12.3		285	76.1		1,504	24.4	
	Fair	596	13.4		46	63		642	17	
	Poor	184	3.3		15	33.3		197	5.6	
Nationality	Saudi	–	–		–	–		6351	12.1	<0.001
	Non-Saudi	–	–		–	–		1925	72.8	

*Based on chi-squared test.

The average number of outpatient visits during the past 12 months by nationality and other characteristics are shown in Table 3. The mean number of visits was significantly higher among Saudis (mean±SD: 1.73 ± 1.351) than non-Saudi (0.92 ± 1.139). The mean value was higher in females (1.68 ± 1.351) compared with males (1.43 ± 1.337). In both Saudi and non-Saudi populations, the elderly group (≥60 years) had higher average health facility visits compared with the adult (26–59 years) and (18–25) groups. The mean values were significantly higher among lower education level in Saudis and non-Saudis (1.64 ± 1.336) than the higher education level (1.54 ± 1.336). In both Saudis and non-Saudis, the mean values of the number of visits were significantly higher among those with high monthly income (+20,000 SR) (1.96 ± 1.453) compared with low-income groups. These were significantly higher among the divorced & widowed (1.83 ± 1.310) than the married and never married groups. The values were found to be higher among those who had GHC (1.69 ± 1.368) and those who had chronic disease (1.92 ± 1.346), which applies to both Saudis and non-Saudis. The average number of visits were significantly lower for those who perceived their general health as being very good (1.40 ± 1.325) compared with those who rated their health as Poor (2.50 ± 1.231). We found similar differences in the number of visits according to the perceived health status group among Saudi and non-Saudi populations.

**Table 3:** Average number of health facility visits for outpatient care during the past 12 months by respondents’ characteristics.

Characteristics	Saudi			Non-Saudi			Total		
	N	Mean±SD	P	N	Mean±SD	P	N	Mean±SD	P
**Sex:**									
Male	3,215	1.71 ± 1.349	0.370^a^	1,330	0.73 ± 1.017	<0.001^a^	4,545	1.43 ± 1.337	<0.001^a^
Female	3,136	1.74 ± 1.351		595	1.33 ± 1.283		3,731	1.68 ± 1.351	
**Age categories:**									
18–25 years	1,593	1.70 ± 1.388	<0.001^b^	319	1.02 ± 1.168	<0.001^b^	1,912	1.59 ± 1.377	<0.001^b^
26–59 years	3,732	1.68 ± 1.338		1,516	0.86 ± 1.111		5,248	1.45 ± 1.330	
≥ 60 years	1,026	1.93 ± 1.324		90	1.50 ± 1.309		1,116	1.89 ± 1.327	
**Education level:**									
Below primary school	1,052	1.91 ± 1.307	<0.001^b^	349	0.84 ± 1.081	.017^b^	1,401	1.64 ± 1.336	0.004^b^
Primary school	495	1.91 ± 1.390		258	0.83 ± 1.127		753	1.54 ± 1.403	
Intermediate school	651	1.76 ± 1.389		357	0.82 ± 0.997		1,008	1.43 ± 1.342	
Secondary school	2,675	1.65 ± 1.352		595	1.00 ± 1.205		3,270	1.53 ± 1.349	
Higher education	1,478	1.66 ± 1.335		366	1.02 ± 1.207		1,844	1.54 ± 1.336	
**Family monthly income:**									
< 5,000	1,433	1.57 ± 1.248	<0.001^b^	1,147	0.70 ± 0.896	<0.001^b^	2,580	1.18 ± 1.186	<0.001^b^
5,000– < 10,000	2,415	1.77 ± 1.357		486	0.98 ± 1.172		2,901	1.63 ± 1.360	
10,000– < 15,000	1,280	1.75 ± 1.398		136	1.60 ± 1.352		1,416	1.74 ± 1.394	
15,000– < 20,000	612	1.70 ± 1.391		69	1.14 ± 1.252		681	1.46 ± 1.387	
20,000+	611	1.92 ± 1.389		87	2.22 ± 1.833		698	1.96 ± 1.453	
**Marital status:**									
Married	3,948	1.68 ± 1.340	<0.002^b^	1,413	0.87 ± 1.109	<0.001^b^	5,361	1.47 ± 1.332	<0.001^b^
Never married	1,986	1.79 ± 1.377		470	0.99 ± 1.197		2,456	1.67 ± 1.380	
Divorced & Widowed	417	1.84 ± 1.322		42	1.67 ± 1.183		459	1.83 ± 1.310	
**Working Status**									
Employed	2,507	1.69 ± 1.365	<0.024^b^	1,466	.85 ± 1.134	<0.001^b^	3,969	1.38 ± 1.348	<0.001^b^
Unemployed	999	1.71 ± 1.367		287	1.29 ± 1.308		1,067	1.69 ± 1.367	
Housewife	2,104	1.73 ± 1.336		391	1.11 ± 1.071		2,493	1.63 ± 1.381	
Retired	747	1.87 ± 1.319		6	1.67 ± 2.066		741	1.86 ± 1.324	
**Type of health coverage**									
Private health insurance	768	1.64 ± 1.265	<0.001^a^	1,402	0.85 ± 1.069	0.001^a^	2,170	1.13 ± 1.204	<0.001^a^
Governmental health coverage	5,583	1.74 ± 1.363		523	1.11 ± 1.290		6,106	1.69 ± 1.368	
**Has chronic diseases:**									
Yes	1,830	2.01 ± 1.351	<0.001^a^	278	1.30 ± 1.135	<0.001^a^	2,108	1.92 ± 1.346	<0.001^a^
No	4,521	1.61 ± 1.335		1,647	0.85 ± 1.127		6,168	1.41 ± 1.326	
**Self-rating of health status:**									
Very good	4,352	1.59 ± 1.336	<0.001^b^	1,579	0.85 ± 1.132	<0.001^b^	5,931	1.40 ± 1.325	<0.001^b^
Good	1,219	1.91 ± 1.329		285	1.11 ± 1.111		1,504	1.76 ± 1.329	
Fair	596	2.07 ± 1.347		46	1.78 ± 1.191		642	2.05 ± 1.338	
Poor	184	2.59 ± 1.226		15	1.40 ± .632		197	2.50 ± 1.231	
**Total**	**6,351**	**1.73 ± 1.351**		**1,925**	**0.92 ± 1.139**		**8,276**	**1.54 ± 1.349**	

**Statistically significant; ^a^ Independent sample t-test for mean difference ^b^ One way ANOVA test.

The regression analysis results examining the factors associated with PHI enrollment are presented in Table 4. The likelihood of having PHI was 27 folds in non-Saudis (OR=27.127; 95% CI = 22.985–32.017) compared with Saudis. Males were found twice the odds to have PHI (OR=2.090; 95% CI = 1.700–2.569) compared with female. Higher-income individuals (15,000–20,000 SR) were approximately twice as likely (OR=1.860; 95% CI, 1.453–2.380) to have the PHI scheme compared with low-income individuals (<5,000 SR). Also, when compared with those with below primary school education level; the odds of having PHI were almost fourfold in those with higher education (undergraduate and above) level (OR=3.584; 95% CI, 2.815–4.563). The likelihood of those who perceive their health as good was two-fold (OR=2.767; 95% CI, 1.350–5.668) higher to have PHI compared with those who perceive their health as Poor. We found that never being married was associated with lower odds compared with being married respectively.

**Table 4:** Factors associated with private health insurance enrollment among the study group.

Variables	Description	Odds ratio (95% CI)
Constant		0.220***
Chronic disease	No (Ref)	1
	Yes	1.068 (0.882; 1.292)
Perceived the health status	Poor (Ref)	1
	Fair	4.141***(1.994; 8.602)
	Good	2.767**(1.350; 5.668)
	Very good	2.343* (1.994; 8.602)
Nationality	Saudi (Ref)	1
	Non-Saudi	27.127*** (22.985; 32.017)
Sex	Female (Ref)	1
	Male	2.090*** (1.700; 2.569)
Monthly income	<5000 (Ref)	1
	5000- < 10000	1.043 (0.881;1.235)
	10000- < 15000	1.166 (0.945;1.438)
	15000- < 20000	1.860***(1.453;2.380)
	20000+	1.426**(1.104;1.841)
Education level	Below primary school (Ref)	1
	Primary school	1.154 (0.882;1.509)
	Intermediate school	1.810*** (1.408;2.327)
	Secondary school	3.197***(2.553;4.002)
	Higher education (undergraduate and above)	3.584***(2.815;4.563)
Age	18-25 years (Ref)	1
	26- 59 years	0.933 (0.705;1.236)
	>60 years	0.815 (0.571;1.163)
Marital status	Married (Ref)	1
	Never married	0.653***(0.541;0.787)
	Divorced &Widowed	0.513***(0.345;0.762)
Log likelihood		−6544.86
Chi-squared		2,977
**N**		**8,276**

Note: *** p < 0.01; ** p < 0.05; * P < 0.10.

The findings from the regression analysis examining factors associated with outpatient healthcare utilization are presented in Table 5. We found PHI enrollment was negatively associated (Coefficient = −0.107; 95% CI: −0.159 to −0.055; P < 0.01) with the utilization of outpatient health services. Individuals with PHI had lower utilization compared with individuals with GHC. Male individuals were less likely to utilize outpatient services compared with females.

**Table 5:** Predictors of the utilization of outpatient care among the study groups.

Variables	Description	Saudi	Non-Saudi	Total
		Co-efficient (95% CI)	Co-efficient (95% CI)	Co-efficient (95% CI)
Constant		0.716***(.584;.848)	0.476(−.027;.979)	0.688*** (0.562; 0.814)
Type of health coverage	governmental health coverage (Ref)	Ref	Ref	Ref
	Private health insurance	−0.059*(−0.119; − 0.001)	−0.101*(−0.213; − 0.011)	−0.107***(−0.159; −0.055)
Nationality	Saudi (Ref)	−	−	0
	Non-Saudi	−	−	−0.392***(−0.456; − 0.329)
Sex	Female (Ref)	0	0	0
	Male	−0.057* (−.115;.001)	−0.506***(−.653; − .358)	−0.142*** (−0.195; −0.089)
Age	18-25 years (Ref)	0	0	0
	26- 59 years	−0.056 (−.015;.127)	−0.187*(−.400;.026)	0.020 (−0.047; 0.086)
	>60 years	−0.027(−.074;.127)	−0.115(−.379;.150)	0.001 (−0.093; 0.094)
Monthly income	<5000 (Ref)	0	0	0
	5000- < 10000	0.176***(.124;.229)	0.282***(.163;.401)	0.230***(0.182; 0.278)
	10000- < 15000	0.188***(.127;.249)	0.656***(.492;.820)	0.275***(0.218; 0.332)
	15000- < 20000	0.164***(.088;.241)	0.308**(.077;.559)	0.228***(0.156; 0.300)
	20000+	0.266***(.192;.340)	0.951***(.776; 1.126)	0.388***(0.321; 0.456)
Education level	Below primary school (Ref)	0	0	0
	Primary school	0.053(−.031;.136)	−0.079 (−.258;.100)	0.053 (−0.022; 0.129)
	Intermediate school	0.011(−.073;.095)	−0.061 (−.227;.104)	0.028 (−0.047; 0.102)
	Secondary school	−0.048(−.124;.028)	0.094 (−.054;.243)	0.025 (−0.041; 0.091)
	Higher education	−0.047 (−.128;.034)	0.044 (−.126;.213)	0.007 (−0.064; 0.078)
Marital status	Married (Ref)	0	0	0
	Never married	0.178***(.118;.237)	0.012(−.127;.152)	0.161***(0.107; 0.215)
	Divorced &Widowed	0.001(−.081;.081)	−0.102(−.385;.181)	0.008 (−0.070; 0.086)
Chronic disease	No (Ref)	0	0	0
	Yes	0.135***(.080;.191)	0.247***(.101;.392)	0.165*** (0.113; 0.217)
Perceived the health status	Poor (Ref)	0	0	0
	Fair	−0.199***(−.307; − .091)	0.330(−.164;.824)	−0.177***(−0.282; −0.077)
	Good	−0.268***(−.374; − .163)	−0.155(−.638;.327)	−0.265***(−0.367; −0.162)
	Very Good	−0.444*** (−.555; − .334)	−0.311 (−.795;.173)	−0.451***(−0.558; −0.345)
Log-likelihood		−10,272.30	−2,386.60	−12,763.90
Chi-squared (DF)		6486.5(6,327)	2231.6 (1,903)	8917.7 (8,251)
**N**		**6,351**	**1,925**	**8,276**

*** p < 0.01; ** p < 0.05; * P < 0.10.

If we compared those individuals with a lower monthly income (<5,000 SR), the utilization of health services was higher among individuals with higher monthly income (20,000 SR or more) (coefficient = 0.388 95% CI = 0.321; 0.456). It was found that never-married individuals have higher healthcare services utilization (Coefficient = 0.161 95% CI = 0.107; 0.215) compared with married individuals. People suffering from chronic conditions had higher utilization of health services compared with people who had no such conditions.

We conducted separate regression models for the Saudi and non-Saudi populations to further examine the association between PHI enrollment and service utilization among these two distinct populations. In both population groups, PHI enrollment was associated with lower utilization of health services.

In addition to the main independent variable (type of health coverage), several control variables in the regression model showed significant associations with outpatient healthcare utilization. Males had lower utilization compared with females. Higher income levels, being never married, having chronic diseases, and had poor health were all associated with higher utilization. These findings highlight the importance of individual characteristics in understanding patterns of healthcare utilization.

Multicollinearity was assessed using the Generalized Variance Inflation Factor (GVIF). All GVIF values were below 5, indicating no evidence of multicollinearity among the independent variables.

## Discussion

The study highlighted the characteristics of PHI enrollees and the association between PHI enrollment and the utilization of outpatient healthcare services in KSA. The results showed that non-Saudi nationality, higher monthly income, male sex, married marital status, higher education level, and good perceived health status were associated with increased PHI enrollment. Further, the factors associated with increased utilization of outpatient services were being Saudi, female, with a high monthly income, never married, having chronic diseases, and a perception of poor health status. Finally, we found that respondents with PHI were significantly associated with lower outpatient service utilization compared with GHC. PHI enrollees in our study were more likely to report favorable health status, which may indicate a lower perceived need for outpatient care. In addition, many PHI enrollees were non-Saudi expatriate workers in the private sector who were required to undergo pre-employment medical check-ups to assess fitness for work, and some PHI arrangements may involve access-related barriers. These factors may partly explain the lower outpatient utilization observed among PHI enrollees.

### Characteristics of private health insurance enrollment

The study revealed a significantly higher proportion of non-Saudis who were covered by PHI. This may be attributed to the implementation of the first phase of the employer-sponsored insurance scheme, which began in 2006 and was fully implemented by 2019, that necessitated compulsory insurance for all those who are working in the private sector, where mostly expatriates are employed [17]. In KSA, about 80% of the workforce in the private sector are non-Saudi expatriates [32]. This pattern may also help explain the large odds ratio observed for non-Saudi nationality in our regression analysis, as PHI eligibility in the pre-reform period was closely linked to employment in the private sector rather than nationality alone. Consistent with this, Al-Hanawi and colleagues reported that 38% of the total population in Saudi Arabia was covered under an employer-mandated insurance scheme, including approximately 2.7 million Saudi citizens, representing about 22% of the PHI-covered population, and around 9.4 million expatriate residents (non-Saudis), accounting for about 78% [20]. The remaining Saudi citizens had free access to public healthcare facilities under GHC and could also use private healthcare facilities on a fee-for-service basis through out-of-pocket payments or by purchasing voluntary PHI [20]. Although Saudi citizens have free access to public healthcare services, some opt for the uptake of PHI to benefit from potential advantages such as avoiding relatively long waiting times and access to a wider range of services, including luxurious services like more appointment options, extra accommodation services, and additional health services [33,34]. Moreover, the GASTAT showed that 21.3% of individuals preferred to visit private healthcare services and paid entirely out-of-pocket for the same reasons [18].

Individuals with higher incomes were approximately twice as likely (OR=1.860) to have PHI compared with low-income individuals, which aligns with the findings of multi-country research in Nigeria, South Africa, and the United States of America [35,36]. One explanation is that self-employed Saudi citizens were less likely to have PHI because the employer-mandated insurance scheme did not cover them prior to 2019. Instead, they relied on free services provided by the GHC, and if they wanted private services, they had to either purchase PHI voluntarily or pay out-of-pocket. This explains their lower enrollment in PHI, particularly among those with lower incomes [25]. Those with lower incomes may be more likely to refrain from enrolling in PHI due to their financial condition [34]. The non-Saudi individuals are required to have PHI irrespective of their employment condition (e.g., employed, self-employed, or unemployed) to stay in the country legally [19].

Males were more likely to have PHI than females, which is in line with a previous study in KSA [37]. This result may be attributed to the workforce composition in Saudi Arabia (79.9% of males working compared with 21.1% of females), which is characterized by male predominance [38,39]. Moreover, considering that employed individuals are compelled to obtain PHI, this could explain the heightened prevalence of insurance coverage among males. In contrast, in most European zhealth coverage system, where public health insurance is mandatory for all residents regardless of employment status [40].

The study showed that the married were more likely to have PHI, which comes in line with the previous study which revealed that being married was associated with a tenfold increase in the likelihood of owning PHI compared with patients who were never married, which suggests that married individuals may be more inclined to secure insurance due to factors like the desire to protect children and mitigate the risk of catastrophic healthcare expenses [41].

The level of education has been identified as a significant factor associated with enrollment in PHI. This finding aligns with the results of a previous study conducted in Ghana [42] and low- and middle-income countries [43]. It is worth noting that individuals with higher levels of education tend to possess a better understanding of the PHI policy compared with those with lower educational backgrounds [44]. Additionally, individuals with a higher educational background have the opportunity to comprehend the fundamental concepts and advantages of the PHI scheme [44]. Moreover, individuals with a higher educational background become more knowledgeable about healthcare issues that are directly associated with the health and well-being of their families [42]. Since higher-educated individuals are more likely to be employed, compared with individuals with low educational qualifications, they have a higher chance of enrolling in PHI through their employment. The high proportion of individuals with PHI who reported good health, which could be attributed partly to the findings in the current study that the majority of insured (PHI) are expatriates, and it is known that expatriates in KSA are mandated to undergo pre-employment medical examinations before they enter the country to ensure their fitness for the job [45].

### Factors associated with outpatient healthcare utilization

The study found that individuals with PHI were less likely to utilize outpatient health services, which is consistent with a previous systematic review by Zhang et al. (2020). This finding challenges the common assumption that insurance coverage enhances access to healthcare services [46]. The majority of the individuals with PHI in our study were non-Saudi (expatriates). Previous research has shown that expatriates with PHI are commonly younger, often in the 30–39 years age group, while the median age of the entire population in Saudi Arabia is 35.5 years [12]. In our study, most PHI enrollees were within the 26–59 years age group, reflecting a predominantly working-age population. These individuals exhibit a relatively low prevalence of chronic ailments and tend to perceive their health as being in good or very good condition (52.8%), resulting in a relatively lower need for health services. Another possible factor, including limited understanding of the insurance policy (i.e., health insurance literacy), may help explain the reasons behind the limited utilization of healthcare services by individuals with PHI [12,47]. Another explanation has been addressed by AlNemer (2018), who notes that expatriates perceive the PHI policy has a weak quality with limited benefits, as it is often done by the employer with the lowest premium [27]. On the other hand, the relatively high utilization by Saudis could be understood from the fact that they have free access to health services through the GHC [27].

Higher monthly income was found to be associated with increased utilization of health services. Al-Hanawi et al. (2021) and Ukert et al. (2022) attribute this positive association to the highest rate of utilization of health services among those with high incomes [48,49]. It has been assumed that “those with better income may be in a position to support themselves in accessing private healthcare services or purchase PHI, which has been found to contribute to easier access to healthcare” [11].

Older age respondents showed a higher average number of outpatient visits in the descriptive analysis (Table 3). Elderly individuals tend to have a higher prevalence of non-communicable diseases, which ultimately leads to greater healthcare utilization [50]. Females are more likely to utilize healthcare services, which is consistent with prior research [51]. However, the association between higher female morbidity and increased healthcare utilization remains inconsistent [52]. Notably, women reported significantly poorer self-perceived health status, which may partially explain their greater use of certain services, such as general practitioner visits and diagnostic procedures [53]. The study of Obuchowska et al. (2020) noted that women of reproductive age frequently visit gynecologists, often seek check-ups for pregnancy tests or contraceptive needs and preventive care, while others visit due to specific health concerns such as pelvic pain or abnormal bleeding [54]. The never-married individuals demonstrated a higher frequency of outpatient healthcare service utilization. Never-married individuals tend to access healthcare services more frequently than married individuals, such as visits to general practitioners, psychiatrists, and psychologists; divorced individuals, in general, also report a higher prevalence of unmet healthcare needs, which is often associated with higher levels of depressive symptoms [55]. Moreover, a large proportion of single females, 73.6% tend to undergo cosmetic surgery to improve their confidence [56].

Having a chronic disease was associated with a significant increase in the utilization of health services, as individuals with chronic conditions need frequent visits for regular check-ups and receive treatment, and they are more susceptible to further complications [57,58]. In KSA, the reports pointed to the relatively high prevalence of preventable chronic diseases such as hypertension, diabetes, and hypercholesterolemia [59]. In a study conducted by Alsubaie et al. in KSA, it had been shown that chronic disease doubles the probability of utilization of healthcare services (OR = 2.02) [60]. The results of the current study showed that the perceived health condition was a substantial factor associated with the utilization of health services with a significantly higher number of visits from those who ranked their health as being “Poor”, which is supported by the theoretical model of health utilization, indicating that the perceived needs for healthcare are a key predictor of utilization of health services [29,61].

### Strengths and limitations

The main strength of the study is based on nationwide data, including a representative sample from all geographic regions of KSA. Further strength could be the application of multiple regression models in this study to examine factors associated with PHI enrollment and its association with outpatient service utilization in KSA while adjusting for confounders. However, due to the cross-sectional nature of the SFHS, one inherited limitation of this study is that it is unable to conclude any causal relationships. In addition, undocumented or irregular migrants may have been underrepresented in the survey data; therefore, the findings may not be fully generalizable to all residents in Saudi Arabia. Another limitation of the study is the potential recall bias in reporting healthcare utilization (1 year) and other information by the respondents. Employment status was not included in the regression analysis due to its close correlation with income, which was already included as a key socioeconomic variable. Also, the categories of captured PHI in the 2018 SFHS include employer-sponsored and individually purchased PHI, whereas other schemes of PHI (e.g., visitor, pilgrim, and domestic worker) were not included in the SFHS. We were able to access only SFHS 2018 data for this study, while a recent version of the dataset was released by the General Authority for Statistics after completion of the analysis. We were unable to include inpatient care utilization in our analysis due to the unavailability of this information in the dataset we received from the GASTAT.

## Conclusions

The study investigated the characteristics associated with enrollment in PHI and outpatient service utilization in Saudi Arabia. It found that being non-Saudis, males, having a high income, a high level of education, and a perceived good health status are more likely to be enrolled in PHI. Factors associated with increased utilization of outpatient services were being female, having a high monthly income, being never married, having chronic diseases, and the perception of poor health. However, PHI was associated with lower utilization of outpatient services. The findings indicate a disparity in healthcare utilization between individuals covered by PHI and those with GHC. Based on these associations, policymakers should take more targeted measures that address the challenges and opportunities associated with PHI enrollment and healthcare service utilization. Introducing targeted interventions aimed at enhancing health insurance literacy and understanding insurance benefits for the insured population could be helpful to improve the current level of health service utilization. These measures could involve creating a robust education campaign and enhancing the accessibility of health services to diverse population groups. To support equitable access to healthcare, there is a need to enhance public awareness and coverage of essential benefits for PHI. Insurance companies should offer flexible and affordable plans to facilitate enrollment by employers and individuals who voluntarily choose to enroll. Future research can focus on evaluating the post-reform impact of PHI in ensuring a diversified type of health services (e.g., including inpatient care) and its effects on quality and health outcomes for entire populations.

## Abbreviations

KSA: Kingdom of Saudi Arabia
SFHS: Saudi Family Health Survey
MOH: Ministry of Health
WHO: World Health Organization
GCC: Gulf Cooperation Council
GASTAT: General Authority for Statistics
PHI: Private Health Insurance
GHC: Governmental Health Coverage
NHI: National Health Insurance
